# PRODUCT REVIEW / ÉVALUATION DE PRODUIT

**DOI:** 10.29173/jchla29916

**Published:** 2026-04-01

**Authors:** Jeff Mason, Jeannie An

**Affiliations:** 1 Health Sciences Librarian McMaster University Hamilton, ON, Canada; 2 Research Services Librarian McMaster University Hamilton, ON, Canada

**Product:** GlobalData Intelligence Center (Medical) (GDIC-M)

**URL:** https://www.globaldata.com/industries/medical-devices/

## Purpose

This product review describes GlobalData Intelligence Center (Medical) (GDIC-M) [1] considering the resource from the intersection of health and business librarianship.

## Product description

GDIC-M is a comprehensive market intelligence tool covering the medical devices and diagnostics industries. It contains information about public and private companies, clinical trial sponsors, investors, marketed and upcoming products, and clinical trials.

According to GlobalData, in-house researchers and analysts use real world data (e.g., hospital purchasing, SEC filings), secondary qualitative and quantitative research (e.g., company websites, scientific journals), primary research (i.e., interviews and surveys), and company financial analyses to inform content, reports, and models. Some information is also gathered using artificial intelligence-based techniques. At the time of writing, GDIC-M includes data from 39 countries, including Canada.

Users are presented with multiple entry points to access data sets, reports, and analyses. Users can navigate directly to company, sector, or country information, access specialized databases, and discover industry and country reports. Each section of GDIC-M allows users to save search results, create alerts, and share results with colleagues who have access to the product.

The remainder of this product review focuses on the content and features of three sections of the resource: Companies, Sectors, and Core Databases.

### Companies

**Fig. 1:**
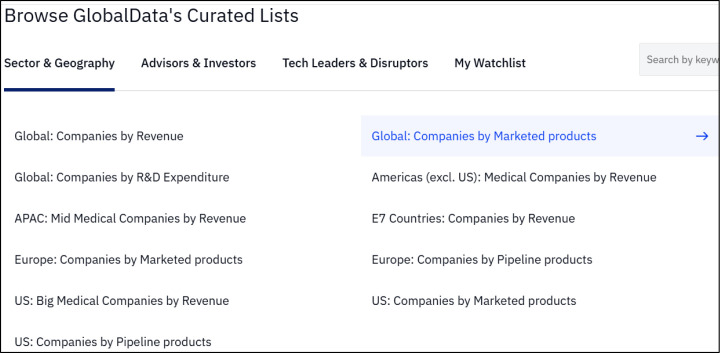
The Sector & Geography curated company lists available in the Companies section of GlobalData Intelligence Center (Medical). Users can also browse curated lists of Advisors & Investors, Tech Leaders & Disruptors, and the user-created My Watchlist.

The Companies section allows users to search for companies or browse curated (Figure 1) or user-created lists of companies.

Clicking any list brings the user to a customizable table where they can filter companies by type (e.g., public, private) or by name. Selecting any company from a list or search results brings the user to its respective information page containing a brief overview, a detailed profile, financial information, peer companies, and a strengths, weaknesses, opportunities, and threats (SWOT) analysis. Company profiles also connect to related data from GDIC-M such as marketed products, pipeline products, clinical trials, deals, and related reports.

### Sectors

**Fig. 2:**
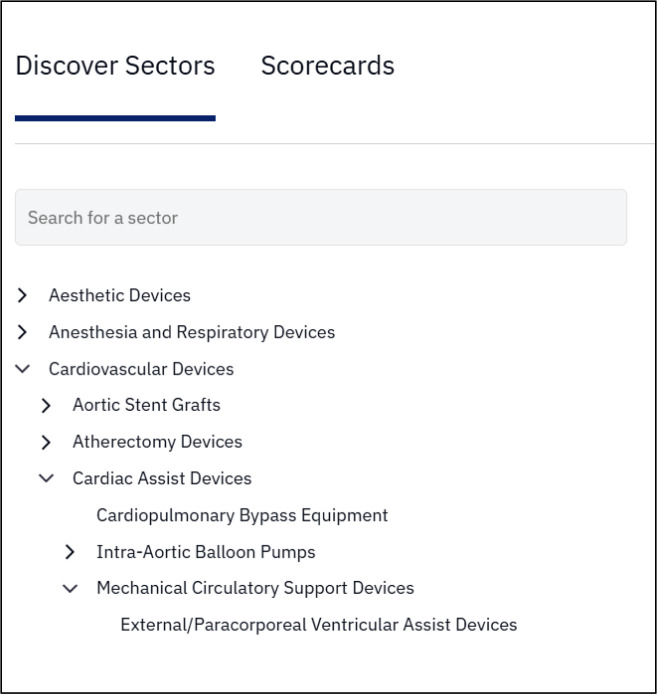
Examples of the sector and sub-sector search and navigation show the hierarchical structure

The Sectors section allows users to explore content about the medical devices industry from different sectors (e.g., cardiovascular devices) or sub-sectors (e.g., thrombectomy catheters) lenses (Figure 2).

**Fig. 3:**
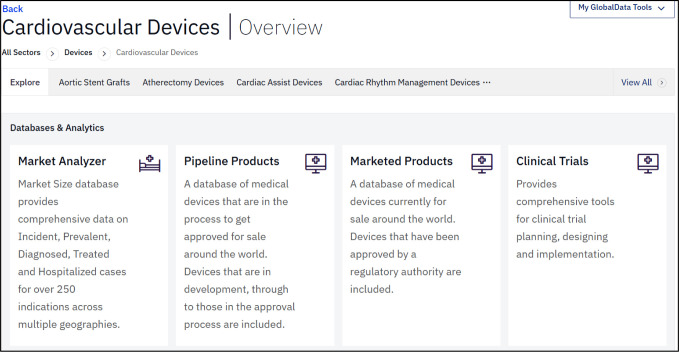
A portion of the cardiovascular devices sector overview page. Users can explore related sub-sectors, or access key databases and other analytics for this part of the medical devices industry.

Each entry presents users with a quick overview of relevant connected data from GDIC-M such as marketed products, pipeline products, clinical trials, deals, and related reports (Figure 3).

Entries may include links to current news, patent analytics, deals, etc. The section also includes “scorecards” intended to “predict the likely leaders and laggards” in the field over the next five years.

### Databases

**Fig. 4:**
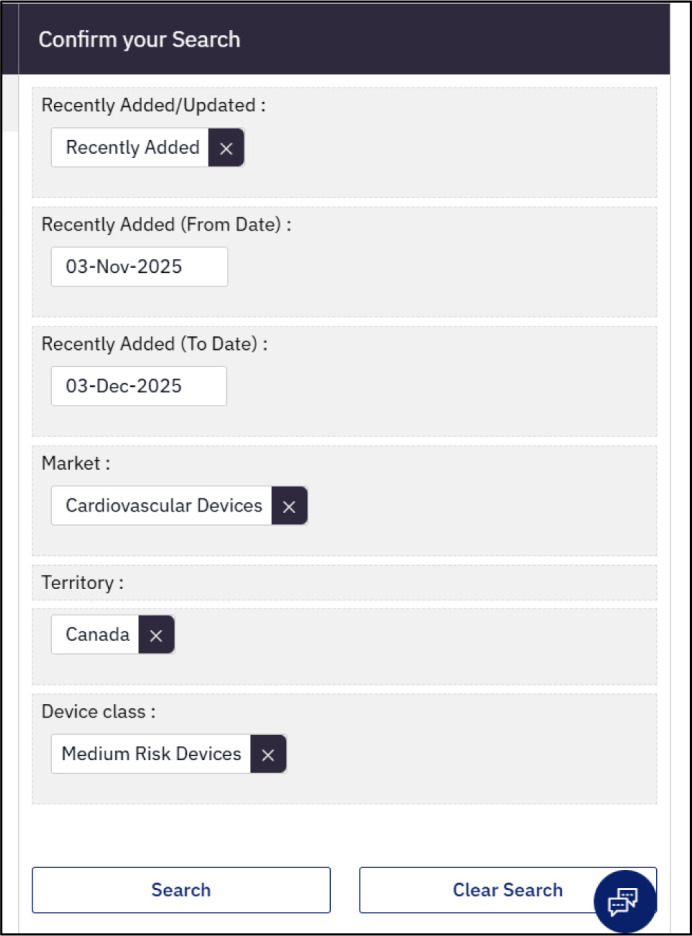
An example of a portion of the Marketed Products database search screen showing some of the options users can choose from when building a search.

The core databases within GDIC-M are Pipeline Products, Marketed Products, and Clinical Trials. At the time of writing, GDIC-M included 17 additional databases containing industry, market, and sentiment-based data (e.g., Social Media Analytics). The core databases use a structured search approach in which users first select a type of information (e.g., device class), then choose from a list of options or enter search terms or date ranges before executing a search (Figure 4). Free text search using Boolean operators is also available.

Search results are presented in tables that can be sorted, filtered, customized, or exported. The “results analytics” option allows users to visualize key aspects of their results (e.g, top 10 therapeutic areas). All results are linked back to related data (e.g., product, company) providing deeper insights and analyses to explore.

### Marketed products

The Marketed Products database allows users to understand the competitive landscape of medical devices and diagnostics currently available on the market. The database covers commercially available products approved since 2010 from 32 countries with an emphasis on products authorized for use in the United States, Australia, Japan, and China. GlobalData states that information is sourced primarily through secondary research from publicly available resources such as company websites, regulatory websites, and conference presentations. Search types include market or equipment type, clinical indication, and device class. Entries in the database contain a product description and specifications. Company, clinical trial, and milestone updates are provided when available.

### Pipeline products

The Pipeline Products database allows users to understand medical devices and diagnostics in development or seeking approval. Search types include stage of development and regulatory pathway. The information in the database is sourced largely from secondary sources such as company websites, clinical trial registries, and investor presentations. Entries in the database contain a product description and product specifications. Company, clinical trial, and milestone updates are provided when available.

### Clinical trials

The Clinical Trials database allows users to search for ongoing and completed clinical trials. Users can search for trials using characteristics of the trial (e.g., indication, study design) or by information about trial sponsors. The information in the database comes from clinical trial registries, news, journal articles and conferences, company websites, and deals reports. Entries in the database contain comprehensive information about each trial.

## Intended audience

GDIC-M may be of interest to organizations supporting health innovation, entrepreneurship, and commercialization through courses, co-curricular activities (e.g., campus accelerators), and technology transfer offices. It may also have uses in organizations involved in early awareness and alert research or health technology assessment to support health care decision-making.

## Special features

Notable features of GDIC-M include:

- “Ask an Analyst” where users can ask asynchronous questions, request custom analyses, and get other help using the resource from a GlobalData analyst
- Proprietary, downloadable, and customizable market models
- Downloadable graphs, charts, and company “storyboards” for use in presentations
- Customizable, user-created watch lists
- Specialized data analyses such as Market Analyzer and Strategic Analysis

## Platform

GDIC-M is web-based and requires no additional software to use most features. Downloading market models, data tables, or company storyboards requires access to software capable working with .xlsx and .pptx files.

## Usability

Users new to market research (e.g., nascent entrepreneurs [2]) may find the interface challenging to navigate at first because of the amount of information and number of access points presented. To support users, GlobalData offers concise video tutorials about various features and functions of the resource.

## Strengths and weaknesses

Strengths of GDIC-M include:

- Breadth and depth of coverage from clinical trial through commercial availability
- Customizable and downloadable market models
- Ability to export data for presentations
- Available support and training
- Cross-linking of data and reports across the platform
- Transparent methodology

Weakness of GDIC-M include:

- Inability to copy and paste text
- Challenging interface that benefits from regular use

## Comparison with similar products

GlobalData offers other industry and cross-industry Intelligence Centres that are relevant to health care audiences including Healthcare, Pharma, and Public Sector versions. GlobalData Explorer also offers coverage of health care industries but without the depth of market data contained in GDIC-M.

Some GlobalData reports are available from ProQuest One Business [3]. At the time of writing, 13 relevant GlobalData publications were available but only five were current. AfterMarket Research (formerly Investext) [4] includes over 1100 GlobalData reports covering the health care industry within the last year.

Other resources that provide some comparable information to GDIC-M include:

- Statista (statistics, reports, topic profiles, market insights, forecasts, surveys) [5]
- IBISWorld (industry reports based on North American Industry Classification System [NAICS]) [6]
- MarketResearch.com Academic (life sciences coverage includes biotechnology, diagnostics, healthcare, medical devices, and pharmaceuticals) [7]

## Currency

According to GlobalData, the company has processes in place to ensure the currency and accuracy of its data. At the time of writing, the company states that:

- Market models are updated every six months and indicate when data points were last updated.
- Marketed products include commercial devices and diagnostic tests approved since 2010.
- Clinical trials include information about trials initiated since 1998.
- Pipeline product records are updated at least every six months to two years and daily following GlobalData’s continuous monitoring processes.

Research methods and data sources are available for review with access points embedded throughout the resource. Content errors can be reported using the Ask an Analyst feature.

## Cost

GDIC-M is subscription-based. Individual, institutional, and enterprise licenses are available. For pricing details, please contact the vendor.

## Contact information

- Website: https://www.globaldata.com/
